# Application of Carbon Materials Derived from Nocino Walnut Liqueur Pomace Residue for Chlorpyrifos Removal from Water

**DOI:** 10.3390/ma18133072

**Published:** 2025-06-28

**Authors:** Milena Zlatković, Rialda Kurtić, Igor A. Pašti, Tamara Tasić, Vedran Milanković, Nebojša Potkonjak, Christoph Unterweger, Tamara Lazarević-Pašti

**Affiliations:** 1 Faculty of Physical Chemistry, University of Belgrade, Studentski Trg 12–16, 11158 Belgrade, Serbia; 20240214@student.ffh.bg.ac.rs (M.Z.); 20200326@student.ffh.bg.ac.rs (R.K.); igor@ffh.bg.ac.rs (I.A.P.); 2 Serbian Academy of Sciences and Arts, Kneza Mihaila 35, 11000 Belgrade, Serbia; 3 VINČA Institute of Nuclear Sciences—National Institute of the Republic of Serbia, University of Belgrade, Mike Petrovica Alasa 12–14, 11000 Belgrade, Serbia; tamara.tasic@vin.bg.ac.rs (T.T.); vedran.milankovic@vin.bg.ac.rs (V.M.); npotkonjak@vin.bg.ac.rs (N.P.); 4 Wood K Plus—Kompetenzzentrum Holz GmbH, Altenberger Strasse 69, 4040 Linz, Austria; c.unterweger@wood-kplus.at

**Keywords:** food waste, valorization, dynamic filtration, regeneration, remediation, sustainable water treatment

## Abstract

This study explores the use of carbon materials derived from Nocino walnut liqueur pomace residue for the removal of chlorpyrifos, a widely used organophosphate pesticide, from water. Carbon adsorbents were synthesized from young walnut biomass under different thermal and chemical treatment conditions, and their structural and surface properties were characterized using BET analysis, FTIR, SEM-EDX, Boehm titration, and zeta potential measurements. The materials exhibited distinct textural and chemical features, including high surface areas and varied surface functionalizations. Batch adsorption studies revealed that the chlorpyrifos removal followed pseudo-second-order kinetics and was best described by the Freundlich and Langmuir isotherms, indicating a combination of pore filling and physisorption via π-π and van der Waals interactions. The highest adsorption capacity of 45.2 ± 0.2 mg g^−1^ was achieved at 30 °C. Thermodynamic analysis confirmed the process to be endothermic, spontaneous, and entropy-driven, with desolvation effects enhancing the performance at elevated temperatures. Dynamic filtration experiments validated the practical applicability of the materials, while moderate reusability was achieved through ethanol-based regeneration. These findings demonstrate the potential of walnut pomace-derived carbons as low-cost, renewable, and effective adsorbents for sustainable water decontamination.

## 1. Introduction

The issue of pesticide contamination in water is a significant and growing environmental concern worldwide [1]. Pesticides are widely used in agricultural practices to control pests and enhance crop yields [2]. However, the extensive use and improper management of these chemicals have led to their persistence in the environment, especially in aquatic ecosystems [3,4]. Chlorpyrifos (CHP), in particular, is highly toxic to aquatic organisms and humans, especially with long-term exposure [5,6]. It is an insecticide that targets the nervous system of pests by inhibiting acetylcholinesterase, an enzyme crucial for nerve function [7,8]. However, CHP is not selective to only harmful insects, and non-target organisms in the environment can also be affected [9,10]. Humans can be exposed to CHP through contaminated water, food, and air. Long-term exposure, even at low levels, has been linked to a range of health issues, including neurological disorders, developmental delays in children, and endocrine disruption [11,12]. Moreover, it has been associated with an increased risk of cancer [3,13].

As environmental pollution continues to rise, there is an increasing emphasis on sustainable solutions for mitigating the effects of toxic contaminants in the environment [14,15]. Traditional methods for pesticide removal often come with high costs and negative environmental impacts [16]. Consequently, there has been a shift towards developing innovative, cost-effective, and eco-friendly alternatives for water decontamination.

Due to its hydrophobicity and low water solubility, CHP presents a significant challenge in water treatment [17]. Various technologies have been explored for its removal, including advanced oxidation processes (AOPs) [18], membrane filtration [19], biodegradation [20,21], and adsorption-based techniques [22]. AOPs, such as ozonation and photo-Fenton processes, can achieve high removal efficiencies but often require complex infrastructure, high energy input, and precise operating parameter control (e.g., pH, light intensity) [23]. Membrane technologies like nanofiltration and reverse osmosis also demonstrate good performances but suffer from high capital and operational costs, membrane fouling, and the need for frequent maintenance [19]. Biodegradation methods utilizing specific microbial strains can be cost-effective and environmentally benign, but they are generally slow and sensitive to operational conditions, such as temperature, pH, and nutrient availability [19]. Among these, adsorption has emerged as one of the most efficient and scalable solutions, offering high removal efficiency, operational simplicity, reusability, and relatively low cost [24].

A promising approach is the use of sustainable porous carbons to remove pesticides from contaminated water sources. There are numerous approaches to synthesizing porous carbon materials, with varying economic viability. However, for large-scale applications, the primary criterion is a balance between price and performance. Thus, producing porous carbon-based adsorbents from a relatively inexpensive precursor using cost-effective methods is especially interesting [25,26,27,28]. Therefore, the development of waste-based adsorbents represents a particularly attractive direction for CHP remediation in water treatment applications.

The valorization of agricultural waste into adsorbent materials is gaining attention due to its potential for sustainable pesticide removal [29,30]. One such underutilized agricultural residue is walnut liqueur pomace, a by-product generated during the production of Nocino, a traditional Italian walnut liqueur. The pomace consists of walnut shells, pulp, and other organic materials that are typically discarded. However, walnut liqueur pomace holds significant potential for valorization. Given its natural carbon content, walnut pomace can be processed into carbon materials suitable for use as adsorbents in environmental applications. Furthermore, the unique properties of walnuts, such as their rich lignocellulosic contents [31], provide an excellent foundation for creating carbon materials with specific functionalities. The main advantage of biomass-derived carbon materials lies in the natural porous structure of the precursor material. The carbonization of biomass leads to the formation of already-porous carbons, which can be further enhanced by applying physical or chemical methods to enhance the porosity [32,33,34]. The resulting carbons, with a large surface area, can adsorb various pollutants, including heavy metals, organic contaminants, and pesticides [35,36]. Furthermore, the chemical composition of the biomass can contribute functional groups to the surface of the carbon material [37,38], which may improve its selectivity toward specific pollutants.

In this work, we aimed to develop and characterize carbon materials derived from walnut pomace, synthesized at 900 °C with and without CO_2_ treatment. The adsorption kinetics of the CHP removal, isotherms, thermodynamic studies, and materials’ performances in dynamic filtration systems were investigated. Additionally, this study evaluated the regeneration potential of these materials using ethanol washing. To the best of our knowledge, this study is the first to explore the potential of walnut liqueur pomace as a precursor for developing adsorbents for pesticide remediation, specifically CHP, from contaminated water. In this way, we can address the dual challenge of agricultural waste valorization and environmental protection, providing sustainable and circular-economy-based solutions for environmental remediation. Moreover, this study provides new insights into the adsorption behavior of CHP, a hydrophobic organophosphate pesticide of significant environmental concern, offering valuable data for the development of efficient materials for the remediation of persistent pollutants in aquatic systems.

## 2. Materials and Methods

### 2.1. Material Synthesis and Physicochemical Characterization

Young walnuts (*Juglans regia*) and walnut liqueur pomace residue obtained after nocino walnut liqueur production were used as precursors for the carbon material synthesis. The walnut pomace had undergone prior exposure to ethanol and saccharose during the liqueur-making process. The preparation process involved immersing 40 young green walnuts (average radius around 2 cm) in 1 L of apple brandy (40% ethanol), to which 400 g of saccharose was added. The mixture was left to macerate under ambient conditions for six weeks. After this period, the liquid phase was separated by filtration, and the remaining solid walnut residue was collected. This residue was then used as the raw material for carbonization. It was not washed before the carbonization since our intention was to valorize this agro-industrial waste as a carbon precursor. Next, the walnut pomace residue was separated by filtration and left to dry at room temperature for 10 d. The dried walnut pomace was oven-dried at 90 °C for 2 h to remove any remaining moisture. Carbonization was performed in an electrical tube furnace (Protherm Furnaces, Ankara, Turkey) under a nitrogen atmosphere. The heating rate was set to 5 °C min^−1^ until the final temperature was reached, followed by an isothermal holding step at 900 °C for 1 h. One sample was subjected to additional treatment using a CO_2_ flow of 100 L h^−1^ (measured under atmospheric pressure and room temperature, corresponding to 68 mmol min^−1^) during the isothermal step for 1 h. For reference, one sample was made using only young walnuts without any ethanol pre-treatment and without CO_2_ treatment after carbonization. Three distinct carbon materials were synthesized (Table 1).

After carbonization, all materials were ground to a fine powder and washed with 0.1 mol dm^−3^ NaOH and HCl and deionized water to remove residual inorganic impurities. Finally, the obtained carbon powders were suspended in 50% ethanol to prepare stock suspensions at a concentration of 2 mg cm^−3^ for further experimental applications. The use of a 50% ethanol–water mixture for preparing stock suspensions was chosen due to the low aqueous solubility of chlorpyrifos (approximately 1.4 mg dm^−3^ at 25 °C) [17]. This solvent system ensured the better distribution and dissolution of chlorpyrifos during the adsorption experiments, allowing for a more reliable and reproducible assessment of the adsorption kinetics and isotherms.

The structural, morphological, and chemical properties of the studied materials were obtained using diverse physicochemical methods. The morphologies and elemental compositions were examined using a PhenomProX scanning electron microscope (SEM) equipped with Energy-Dispersive X-ray Analysis (EDX), both from Thermo Fisher Scientific, Waltham, MA, USA.

Fourier-transform infrared (FTIR) spectroscopy was performed using a Nicolet iS20 FT-IR spectrophotometer (Thermo Fisher Scientific, Waltham, MA, USA) to identify the samples’ functional groups and molecular bonds. Spectra were collected over the wavenumber range of 4000 to 400 cm^−1^, with 64 scans per measurement at a resolution of 4 cm^−1^.

Zeta potential (ZP) measurements were conducted using a Nano ZS Zetasizer system (Malvern Instruments, Malvern, UK) equipped with a 633 nm He-Ne laser to assess the surface charge characteristics of the materials.

The specific surface areas (S_BET_), pore volumes (V_tot_), average pore diameters (d_av_), and pore size distributions (PSDs) were determined via nitrogen adsorption–desorption isotherms at −196.15 °C using an Autosorb-iQ gas sorption system (Anton Paar QuantaTec Inc., Graz, Austria). Specific surfaces were evaluated using the BET method [39], while PSDs were evaluated using the non-local density functional theory (NLDFT) model. Before analysis, samples were degassed under a vacuum at 300 °C for 5 h to remove any adsorbed impurities, ensuring an accurate assessment of the materials’ textural properties.

To quantify the oxygen-containing functional groups on the material surface, Boehm titration was employed. A 10 mL suspension of the material at a concentration of 1 mg/mL was titrated with 10 μL increments of 0.01 mol/dm^3^ solutions of NaOH and 0.005 mol/dm^3^ solutions of NaHCO_3_ and Na_2_CO_3_. Sodium bicarbonate reacts selectively with carboxyl groups, sodium carbonate neutralizes both carboxyl and lactone groups, while sodium hydroxide targets carboxyl, lactone, and phenol groups. The amounts of each functional group were determined based on the differences in the volumes of titrant consumed. pH measurements were performed using a Metrohm 713 pH meter (Herisau, Switzerland) equipped with a combined electrode to ensure accuracy.

### 2.2. Adsorption Studies and Material Regeneration

Adsorption experiments were conducted under stationary (batch) and dynamic (in a filter) conditions. Batch adsorption experiments were performed to evaluate the interaction between CHP and the synthesized carbon materials. CHP is a hydrophobic, neutral organophosphate pesticide with low aqueous solubility (1.4 mg dm^−3^ at 25 °C). Its maximum lateral dimension is approximately 10.75 Å [40]. For each experiment, 0.5 cm^3^ of stock dispersions of the selected adsorbents (2 mg cm^−3^, pH 6) was mixed with 0.5 cm^3^ of CHP solutions prepared in 50% ethanol–water mixtures at varying concentrations. The mixtures were held on a laboratory shaker for predetermined time intervals. Following adsorption, the samples were centrifuged at 14,500 rpm, and the supernatant was filtered using a nylon membrane filter to remove any residual particles.

The concentration of CHP in the filtrate was determined via Ultra-Performance Liquid Chromatography (UPLC) using a Waters ACQUITY UPLC system equipped with a Photodiode Array (PDA) detector and managed by Empower version number 3 software. A BEH C18 column (1.7 μm, 100 mm × 2.1 mm) was used under isocratic conditions with a mobile phase comprising 20% water with 10% acetonitrile and 80% pure acetonitrile. The flow rate was set at 0.2 cm^3^ min^−1^, with an injection volume of 5 μL. CHP detection was carried out at 200 nm. Control experiments without adsorbent were conducted to ensure accuracy.

Adsorption experiments were performed under environmentally relevant conditions: −20, 25, and 30 °C, pH 6, and CHP concentrations ranging from 5 × 10^−6^ to 5 × 10^−4^ mol dm^−3^ to simulate potential real-world applications. Each experiment was repeated in triplicate, and mean values with error bars representing the highest deviations were reported.

Kinetic studies were conducted by mixing 1 mg cm^−3^ of each material with 5 × 10^−5^ mol dm^−3^ of CHP at 20 °C, with contact intervals ranging from 1 to 90 min. The CHP concentrations were quantified using UPLC, and the amount adsorbed was calculated by subtracting the remaining CHP from the initial concentration. Data were fitted to non-linear pseudo-first-order (PFO), pseudo-second-order (PSO), Elovich, and intraparticle diffusion (IPD) models. All model equations are provided in the Supplementary Materials (Table S1).

For the adsorption isotherms, 1 mg cm^−3^ of material was incubated with CHP solutions at concentrations from 5 × 10^−6^ to 5 × 10^−4^ mol dm^−3^ for 60 min at 20, 25, and 30 °C. The isotherm data were analyzed using the Freundlich, Langmuir, Temkin, and Dubinin–Radushkevich models. All model equations are provided in the Supplementary Materials (Table S2).

The dynamic adsorption behavior was examined using modified commercial syringe filters. Each carbon material (1 mg) was dispersed in 1 cm^3^ of 50% ethanol and introduced into a nylon syringe filter (220 nm pore size, KX Syringe Filter, Kinesis, Cole Parmer, St. Neots, UK). Compressed air was applied to expel excess water before introducing the pesticide solution. Subsequently, 1 cm^3^ of CHP solution (5 × 10^−5^ mol dm^−3^) was passed through the modified filter at a constant flow rate of 1 cm^3^ min^−1^. The effluent was collected and analyzed via UPLC to determine the amount of CHP removed.

Regeneration of the adsorbents was carried out by washing the modified filters with 5 cm^3^ of 96% ethanol for 1 min. This process was repeated to assess the reusability and stability of the adsorbent materials over multiple adsorption–desorption cycles.

### 2.3. Toxicity Assessment

Given that organophosphate pesticides are well-known inhibitors of acetylcholinesterase (AChE), the toxicity of CHP before and after contact with the adsorbents was evaluated by measuring the AChE inhibition. A modified Ellman’s assay [41,42] was applied, in which 2.5 IU of commercially available AChE (from electric eel, Sigma Aldrich, Taufkirchen, Germany) was pre-incubated with CHP solutions for 20 min in 50 mM phosphate buffer (pH 8.0) at 37 °C. The enzymatic reaction was initiated by adding acetylthiocholine iodide (AChI) (0.075 mol dm^−3^) and 5,5′-dithiobis-(2-nitrobenzoic acid) (DTNB) (1 × 10^−4^ mol dm^−3^) as the chromogenic reagent (both from Sigma Aldrich). After 8 min of reaction, the process was halted using 10% sodium dodecyl sulfate (SDS). The reaction product, thiocholine, reacts with DTNB to form 5-thio-2-nitrobenzoate, the absorbance of which was measured at 412 nm. The intensity of the resulting yellow color served as a direct indicator of enzyme activity. The enzyme concentration was kept constant to ensure a stable and reproducible spectrophotometric signal. The percentage of AChE inhibition, reflecting the toxicological impact of the solution, was calculated according to the following equation:

EI = 100 × (EA_0_ − EA)/EA_0_

where EA_0_ denotes the enzyme activity in the absence of CHP, and EA is the activity after exposure to the pesticide.

## 3. Results and Discussion

### 3.1. Insight into the Physicochemical Nature of the Materials

The BET analysis revealed notable differences in the specific surface areas and total pore volumes among the synthesized carbon materials (Table 2). W900 exhibited the lowest specific surface area and total pore volume, indicating a more compact structure with fewer accessible pores. In contrast, WLP900 demonstrated a significant increase in both parameters, with a specific surface area of 737 m^2^ g^−1^ and a total pore volume of 0.332 cm^3^ g^−1^, suggesting that the presence of saccharose from the liqueur and ethanol impregnation provided additional carbon sources, facilitating the development of a more extensive pore network. Interestingly, the CO_2_ treatment did not further increase the surface area as expected. Instead, WLP900CO_2_ exhibited slight reductions in both its specific surface area and total pore volume. While these decreases are relatively small, they suggest that the CO_2_ treatment may have caused partial pore collapse or restructuring rather than additional pore formation. This outcome is somewhat unusual, as CO_2_ treatment typically enhances porosity [43]. However, the results indicate that the effects of CO_2_ treatment may be highly dependent on the initial material composition and the specific conditions of the synthesis process. In contrast, the effects of CO_2_ treatment do not necessarily have a positive effect on pore development [44]. As sample W900 had much lower S_BET_ and V_tot_ values compared to the other two samples, which could be attributed to the ethanol washing and the presence of saccharose in the precursor, we further focused on the characterization and application of samples WLP900 and WLP900CO_2_.

In a recent study by Serafin et al. [45], activated carbons derived from walnut shells using KOH chemical activation exhibited extremely high surface areas (up to 1868 m^2^ g^−1^) and micropore volumes (0.94 cm^3^ g^−1^). While the surface areas of our carbon materials are notably lower, our approach offers several practical and environmental advantages. It includes a safer and more sustainable preparation method without the use of corrosive activating agents. Furthermore, our materials were developed specifically for aqueous pesticide removal and showed high adsorption performances for chlorpyrifos without chemical activation.

**Figure 1:**
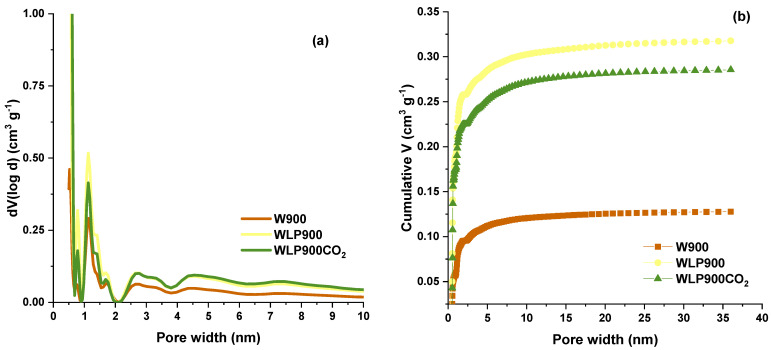
(**a**) Pore size distributions of the studied samples; (**b**) cumulative pore volumes of the studied samples as a function of the pore width.

While the total pore volumes and specific surfaces give an overall indication of the sample porosity, the PSD curves (Figure 1a), derived from the N_2_ adsorption isotherms (Figures S1–S3, Supplementary Materials), show that the samples are dominantly microporous with similar PSDs, differing primarily in magnitude for pores below 2 nm in diameter, while the magnitude follows the same trend as that of the S_BET_. In contrast, the average pore diameter follows the opposite trend to that of the S_BET_ (Table 2). The PSD curves for WLP900 and WLP900CO_2_ practically overlap for pores with d > 2 nm. In line with the microporous nature of the studied carbons, over 90% of the V_tot_ is contained within the pores, which are below 4 nm in diameter (Figure 1b). The same applies to the specific surfaces (Figure S4, Supplementary Materials), showing that practically the entire S_BET_ is within the pores smaller than 5 nm. This leads to the conclusion that the external surface is exceptionally small, and the differences in the adsorption behavior are due to the differences in the pore distribution for pores below 5 nm.

Further characterization and adsorption experiments were conducted with the carbon materials derived from walnut liqueur pomace (WLP900 and WLP900CO_2_), while W900 was not considered further due to the much lower specific surface area and pore volume.

**Figure 2:**
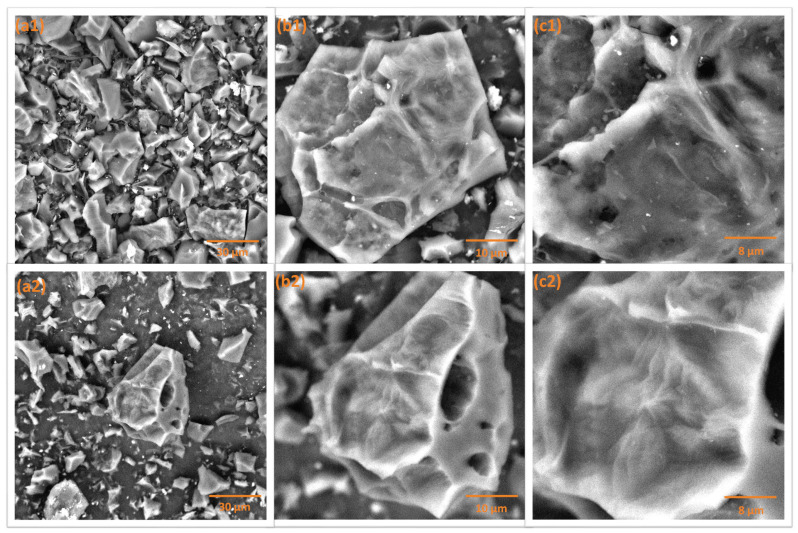
SEM visualization of the investigated carbon materials: WLP900 (**top row**) and WLP900CO_2_ (**bottom row**) at magnifications of (**a1**,**a2**) 2000×, (**b1**,**b2**) 5000×, and (**c1**,**c2**) 10,000×.

The carbon samples’ surface structures were visualized via SEM under three distinct magnifications (Figure 2). The micrographs reveal that all three samples exhibit similar morphologies with no significant visible differences. The surfaces appear rough and uneven, with clearly distinguishable pores. Additionally, the edges of the structures appear sharp, further indicating the textural characteristics of the materials.

In contrast to the similarities observed in the surface morphologies, the EDX results reveal significant differences in the elemental compositions of the investigated materials. WLP900 exhibits the highest carbon content (85.35 at.%), indicating a high degree of carbonization. In contrast, WLP900CO_2_ has a notably lower carbon content (68.66 at.%), suggesting a possible effect of the CO_2_ treatment on the material’s structure. The oxygen content in WLP900CO_2_ is rather high (18.48 at.%), which may indicate increased surface oxidation or the formation of oxygen-containing functional groups. Nitrogen is also more abundant in WLP900CO_2_ (11.60 at.%) compared to WLP900, which could enhance adsorption properties through potential interactions with pollutants. Minor elements such as K, Mg, P, Ca, Na, S, Cl, Si, and I vary among the samples, with WLP900 generally containing higher concentrations of these elements than WLP900CO_2_. This variation likely originates from differences in the synthesis conditions and potential mineral retention during processing, as well as the inherent compositional variability of the walnut precursor. Detailed elemental compositions are presented in Table 3.

The FTIR spectra of the tested materials (Figure S5) do not exhibit pronounced absorption bands that can be attributed to specific functional groups. This observation is in line with expectations, given that the materials were carbonized at a high temperature (900 °C) [46]. The severe thermal treatment likely resulted in the decomposition of most organic functionalities, leading to a predominantly carbonaceous structure with only minimal residual functional groups. As a consequence, the characteristic vibrational modes typically associated with functional groups are absent or significantly diminished. Additionally, treatment of the precursor with ethanol and saccharose and further CO_2_ treatment did not induce any observable changes in the presence of functional groups.

Zeta potential measurements were carried out on all the examined materials at 0.5 mg cm^−3^ concentrations to assess their surface charges and determine their isoelectric points. The titrations were performed across a pH range of 1 to 14, using HCl and NaOH as titrating agents. All materials had a starting pH of 6 in their initial stock solutions. As shown in Figure S6 (Supplementary Materials), the isoelectric points of WLP900 and WLP900CO_2_ were determined to be pH 4.3 and 3.0, respectively. Since these two materials initially exhibited a pH of 6 in stock solutions, this suggests that their surfaces carry a negative charge in 50% ethanol.

**Figure 3:**
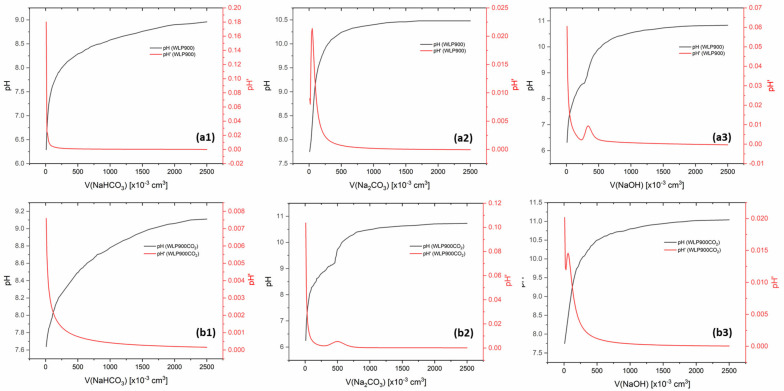
The results of the Boehm titration of (**a**) WLP900 and (**b**) WLP900CO_2_ using NaHCO_3_ (**a1, b1**), Na_2_CO_3_ (**a2, b2**), and NaOH (**a3, b3**).

In addition to FTIR analysis, surface functional groups were quantified using Boehm titration (Figure 3). No carboxyl groups were detected on the surface of either material, as confirmed by the absence of a significant pH shift during NaHCO_3_ titration. In the case of WLP900, the titration with Na_2_CO_3_ reached the equivalence point at pH 6.22 after the addition of 5.1 × 10^−4^ dm^3^ of titrant, corresponding to a lactone group concentration of 2.5 × 10^−4^ mol g^−1^ on the material surface. Subsequent titration with NaOH reached the equivalence point at pH 6.94, indicating a total of acidic groups (lactones and phenols) equal to 3.3 × 10^−4^ mol g^−1^. By subtracting the lactone contribution, the concentration of phenol groups in 1g of the material was calculated as 8.0 × 10^−5^ mol g^−1^.

For WLP900CO_2_, a similar pattern was observed. The Na_2_CO_3_ titration yielded a lactone group concentration of 3.0 × 10^−5^ mol g^−1^, while the NaOH titration indicated a total of 4.8 × 10^−5^ mol g^−1^ acidic groups. After subtracting the lactone contribution, the phenol group concentration in 1 g of the material was determined to be 1.8 × 10^−5^ mol g^−1^.

Although the EDX analysis showed a higher overall oxygen content in WLP900CO_2_ compared to that in WLP900, Boehm titration revealed a lower concentration of surface acidic functional groups. This discrepancy arises from the fundamental differences between the two techniques: EDX measures the total elemental composition near the surface, including both reactive and non-reactive oxygen species, as well as the oxygen incorporated into inorganic residues or within the carbon matrix. In contrast, Boehm titration selectively quantifies only surface-accessible acidic groups that can react under aqueous conditions. Therefore, the higher oxygen content observed by EDX may be due to non-titratable oxygen species or subsurface oxygen not involved in surface adsorption processes. The more negative isoelectric point of WLP900CO_2_, despite its lower Boehm acidity, can be attributed to the presence of non-titratable oxygen groups, such as carbonyls, ethers, or epoxides, introduced during CO_2_ treatment. These groups do not react in Boehm titration but can still contribute to surface polarity and negative charge in aqueous suspensions, thereby lowering the isoelectric point.

The presence of saccharose and ethanol during the pre-treatment of WLP900 and WLP900CO_2_ unambiguously influenced their physicochemical properties, potentially enhancing their suitability for adsorption applications. The zeta potential measurements also indicate that these materials possess a favorable surface charge profile, which may facilitate interactions with pollutants. Finally, the selection to utilize biomass waste from liqueur production aligns with the principles of sustainable waste valorization, contributing to carbon sequestration and environmental remediation.

### 3.2. Adsorption Experiments Under Static Conditions

#### 3.2.1. Adsorption Kinetics

**Figure 4:**
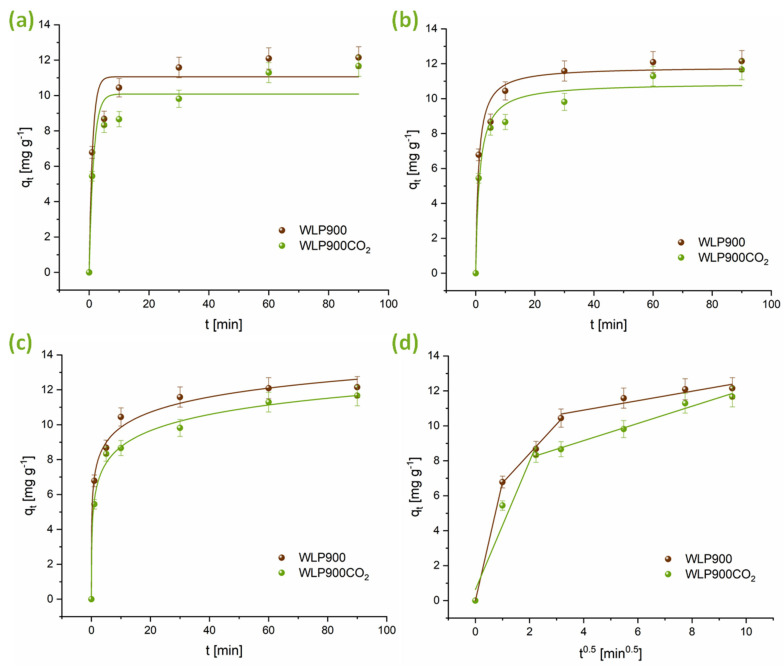
Model fitting of CHP adsorption kinetics using (**a**) PFO, (**b**) PSO, (**c**) Elovich, and (**d**) intraparticle diffusion models.

The adsorption kinetics of CHP on the synthesized carbon materials was evaluated by applying non-linear forms of the PFO, PSO, Elovich, and intraparticle diffusion models. The kinetic profiles are illustrated in Figure 4, while the corresponding model parameters are summarized in Table 4. As shown in Figure 4, adsorption equilibrium was achieved within 60 min. According to the data in Table 4, the PSO model exhibited the best agreement with the experimental results across all the tested conditions. The kinetic rate constants (k2) further reveal that the adsorption of CHP on WLP900 was slightly faster than that on WLP900CO_2_. This difference may be attributed to variations in the SBET, pore structure, and availability of active adsorption sites, which enhance the interaction of CHP with WLP900. Additionally, the Elovich model parameters further support the observed adsorption behavior, where the initial adsorption rate (α) is greater than the desorption constant (β) for both materials, indicating a strong interaction between CHP and the investigated carbon surfaces. The α value for WLP900 is significantly higher than that for WLP900CO_2_, supporting the conclusion that adsorption on WLP900 occurs more rapidly.

The intraparticle diffusion model occurred through three phases for the CHP adsorption onto WLP900. The first phase corresponded to the diffusion of CHP molecules from the bulk solution to the outer surfaces of the investigated materials. The second phase signified intraparticle diffusion, where the molecules penetrated the materials’ pores. The third phase involved the attainment of equilibrium. After each breakpoint, the k_id_ values decreased, suggesting gradually slower adsorption rates. Meanwhile, the increase in the C value highlights the boundary layer’s growing influence in the CHP adsorption process onto WLP900. In contrast, the adsorption of CHP onto WLP900CO_2_ was characterized by two phases.

#### 3.2.2. Adsorption Isotherm Studies

**Figure 5:**
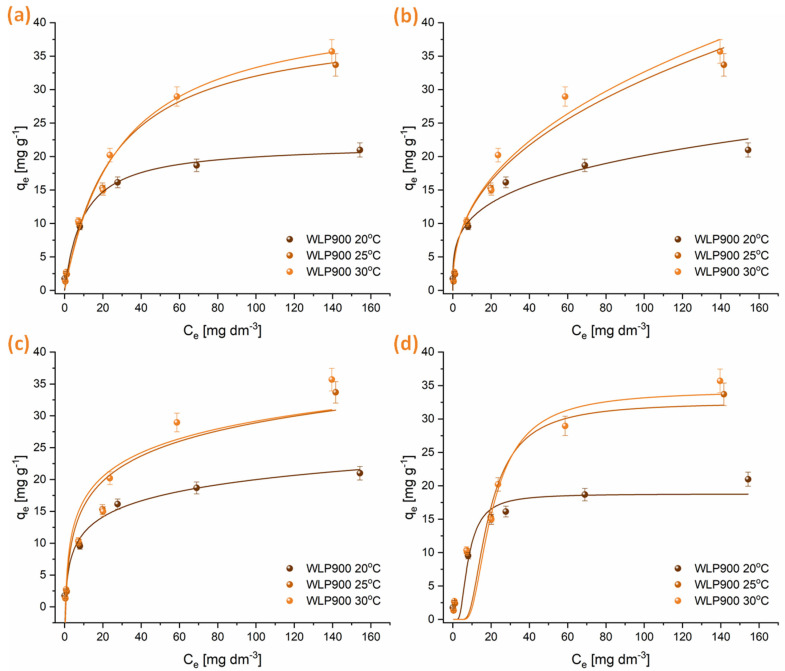
Graphical illustrations of CHP adsorption onto WLP900 at 20, 25, and 30 °C modeled by Langmuir (**a**), Freundlich (**b**), Temkin (**c**), and Dubinin–Radushkevich (**d**) isotherms.

**Figure 6:**
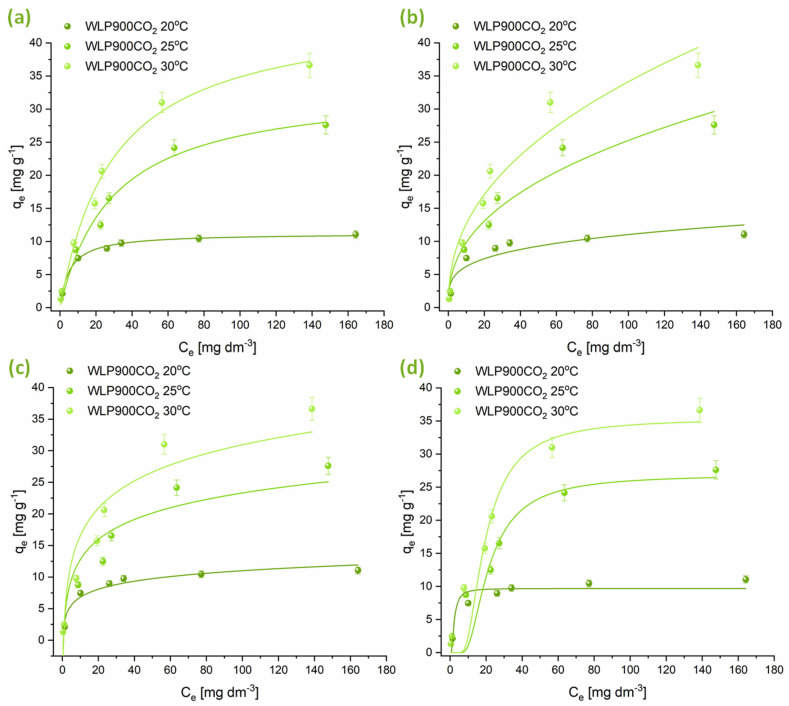
Graphical illustrations of CHP adsorption onto WLP900CO_2_ at 20, 25, and 30 °C modeled by (**a**) Langmuir, (**b**) Freundlich, (**c**) Temkin, and (**d**) Dubinin–Radushkevich isotherms.

Isotherm adsorption experiments were performed at three temperatures, applying the Freundlich, Langmuir, Temkin, and Dubinin–Radushkevich models. The findings are illustrated in Figure 5 and Figure 6, with the relevant adsorption parameters compiled in Table 5. Analysis of the data suggests that the adsorption behavior of CHP on the tested materials is well represented by both the Freundlich and Langmuir isotherms. However, the Langmuir isotherm exhibits a slightly better correlation. The n parameter, obtained from the Freundlich isotherm, indicates that CHP adsorption was a favorable process at all temperatures (n > 1). Nevertheless, the degree of favorability decreased with increasing temperature. The maximum amount of CHP that can be adsorbed per gram of material, derived from the Langmuir isotherm, shows that at lower temperatures, the material that was not physically activated with CO_2_ (WLP900) had a higher adsorption capacity. Still, with increasing temperature, the adsorption capacities of WLP900 and WLP900CO_2_ gradually equalized. Moreover, the CO_2_-activated material exhibited a slightly higher adsorption capacity at higher temperatures, reaching maximum values of 42.9 ± 0.2 mg g^−1^ for WLP900 and 45.2 ± 0.2 mg g^−1^ for WLP900CO_2_. In addition to the adsorption capacities, the Langmuir constant (K_L_), which reflects the affinity between the adsorbate and adsorbent, provides further insight into the interaction strength. At 20 °C, the K_L_ for WLP900CO_2_ was nearly twice as high as that for WLP900, suggesting a stronger initial binding affinity of CHP molecules for the CO_2_-activated surface. This could be attributed to the enhanced accessibility of active sites and the greater pore exposure due to activation. However, as the temperature increased to 25 °C and 30 °C, the K_L_ values for both materials became similar, indicating a temperature-induced leveling of the adsorption affinities. This convergence may have resulted from thermally driven changes in the surface hydration, diffusion dynamics, or adsorbate rearrangement that reduced the advantage of the CO_2_-activated structure at higher temperatures.

The parameters obtained from the Temkin isotherm suggest a decrease in the interaction between the adsorbate and the adsorbent with increasing temperature, which is further confirmed by the reduction in the E parameter obtained from the Dubinin–Radushkevich isotherm. This indicates that CHP adsorbed on the surfaces of these materials by physisorption—weak van der Waals interactions and electrostatic π-π interactions between aromatic moieties of pesticide molecules and the investigated materials. It is particularly interesting to note that at temperatures of 25 and 30 °C, the adsorption capacity of WLP900 exhibits only a slight variation. This trend is evident both in the graphical representations and across all the analyzed isotherms, suggesting that a further increase in temperature will not lead to significant changes in the adsorption capacity of this material (Figure 5). In contrast, in the case of the CO_2_-activated material (WLP900CO_2_), a continuous increase in the maximum amount of CHP adsorbed per gram of material is observed with the rising temperature (Figure 6). This indicates that CO_2_ treatment enhances the thermal stability of active sites, enabling more efficient CHP adsorption even at elevated temperatures.

The superior adsorption performance of WLP900, particularly at lower temperatures, can be attributed to its higher specific surface area and total pore volume, as revealed by BET analysis, along with its greater concentration of surface acidic groups determined by Boehm titration. Its micropore distribution provides enhanced accessibility and stronger interactions with CHP molecules. Namely, the PSD shows a high concentration of pores below 4 nm, which aligns well with the size of the CHP molecule, allowing for its accommodation into the pores [47]. In contrast, WLP900CO_2_ exhibits a slightly lower surface area and fewer acidic groups but possesses a more negatively charged surface and higher oxygen and nitrogen contents, which becomes advantageous at elevated temperatures due to enhanced desolvation and increased adsorbate affinity. These complementary characteristics explain the temperature-dependent shift in the adsorption efficiencies between the two materials.

To evaluate the performances of the obtained materials, their adsorption capacities were compared with the literature-reported values for various biochars used for chlorpyrifos removal. From the results presented in Table 6, it is obvious that the performances of the materials investigated in this paper are comparable to those reported in the literature.

#### 3.2.3. Thermodynamic Parameters

The thermodynamic aspects of the adsorption process were also examined. All equations are provided in the Supplementary Materials. Figure S7 presents the Van’t Hoff plots for the CHP adsorption on all the materials, while Table 7 summarizes the calculated parameters along with their corresponding R^2^ values.

By analyzing the presented Van’t Hoff plots and the obtained thermodynamic parameters, a clear distinction can be observed between the adsorption of CHP onto the investigated materials, which agrees with the previously discussed findings. Both adsorption processes exhibit an endothermic nature, as evidenced by the positive values of the ΔH°, indicating that the adsorption capacity increases with rising temperature (Table 7).

Furthermore, the increase in the system randomness during adsorption, as confirmed by the positive values of the ΔS^o^, indicates an entropy-driven process that leads to increased disorder at the solid–liquid interface. This is particularly relevant for the WLP900CO_2_ material, whose higher oxygen and nitrogen contents (as revealed by EDX) and more negative zeta potential suggest strong solvation in polar media. The pronounced solvation at lower temperatures likely shields adsorption sites, limiting accessibility to hydrophobic chlorpyrifos molecules. As the temperature increases, the desolvation becomes more favorable, exposing active sites and enhancing adsorbate–adsorbent interactions, which explains the improved adsorption performance observed at elevated temperatures. These findings align with the physicochemical characteristics of the materials. While WLP900 benefits from a higher surface area, pore volume, and density of surface acidic groups, leading to a better performance at lower temperatures, WLP900CO_2_ gains an advantage at higher temperatures due to its thermally enhanced desolvation and greater affinity for CHP. The increasingly negative values of the ΔG^o^ with rising temperature confirm the spontaneous nature of the adsorption process for both materials and further highlight the distinct but complementary mechanisms governing their performances.

A notable difference between the two materials is reflected in the thermodynamic parameters. The CO_2_-activated material (WLP900CO_2_) exhibits higher values of both the ΔH° and ΔS° compared to those of WLP900, further supporting its enhanced adsorption performance at elevated temperatures. This aligns with the previously observed trend of a more pronounced increase in the q_max_ with rising temperature for WLP900CO_2_, highlighting the higher affinity and improved adsorption potential of this material.

### 3.3. Dynamic Adsorption Testing and Regeneration Evaluation

The adsorption performances of both materials were evaluated under dynamic conditions using a filtration setup with a contact time of 1 min and an initial CHP concentration of 5 × 10^−5^ mol dm^−3^. The results were compared to those obtained under static conditions. For WLP900, the adsorption under dynamic conditions reached around 30%, while it was 40% under static conditions. For WLP900CO_2_, the adsorption under dynamic conditions was 30%, almost identical to that under static conditions. These comparable values indicate that both materials can be effectively used in filtration systems.

The regeneration of the materials was performed using 96% ethanol. However, after the first regeneration cycle, significant drops in the adsorption efficiencies were observed (Figure S8). Under dynamic conditions, the uptake of WLP900 decreased to 11%, while for WLP900CO_2_, it dropped to 7%. Notably, no further declines in the CHP uptake were observed in the following four regeneration cycles. This suggests that the initial regeneration step led to partial pore blockage, making a certain number of adsorption sites inaccessible in the subsequent adsorption cycles. Despite the significant reduction in the adsorption capacity after regeneration, the remaining capacity (~10 mg g^−1^) was still considerable and remained stable over five cycles. Compared to our previous experience with regenerating biomass-derived carbon materials [52], this regeneration process proved less effective. Therefore, future studies will also explore alternative regeneration strategies to enhance the material reusability, such as the usage of different solvents, heat treatments, or their combinations. Finally, it is important to mention that even with incomplete regeneration, these materials possessed similar or higher adsorption capacities towards CHP in several consecutive regeneration cycles compared to the reported adsorption capacities of different carbon materials found in the literature [53,54,55].

Although physisorption is generally associated with reversibility, the limited regeneration efficiency observed after ethanol washing is not necessarily a contradiction. In this case, the physisorption of chlorpyrifos likely involves pore filling in narrow micropores and weak non-specific interactions (e.g., π-π stacking, van der Waals forces), as indicated by the isotherm model fitting and low Boehm acidity. Once adsorbed, the large, hydrophobic chlorpyrifos molecules may become trapped within ultramicropores (<0.7 nm), where solvent diffusion is hindered, and desorption becomes kinetically limited, even if thermodynamically reversible. This entrapment effect, common in microporous carbons, leads to irreversible physisorption, which ethanol cannot fully overcome in a single short regeneration cycle. Thus, while the adsorption is not governed by strong chemisorption, the physical confinement and low desorption rate explain the regeneration loss, linking the observed behavior to both the pore structure and the nature of the interaction.

### 3.4. Assessment of CHP Toxicity

To evaluate the residual toxicity of CHP after adsorption, we used an Ellman colorimetric assay (Section 2.3). A solution containing 5 × 10^−5^ mol dm^−3^ CHP exhibited 88% inhibition of AChE activity, confirming the pronounced neurotoxic potential of the pesticide. Following treatment with the carbon materials, a noticeable decrease in AChE inhibition was observed. Specifically, after contact with WLP900, the inhibition dropped to 53%, while the treatment with WLP900CO_2_ resulted in 60% inhibition. These reductions indicate a substantial decrease in CHP toxicity, reflecting the effectiveness of the adsorbents in mitigating its toxicological impact. Notably, the decrease in enzymatic inhibition after adsorption suggests that no more toxic by-products, such as chlorpyrifos-oxon, were formed during interaction with the adsorbents. These results confirm that both WLP900 and WLP900CO_2_ reduce the neurotoxicity of chlorpyrifos in treated water, illustrating their potential for safe and efficient water remediation applications.

## 4. Conclusions

This study demonstrated the potential of carbon materials derived from Nocino walnut liqueur pomace residue for the removal of chlorpyrifos (CHP) from aqueous solutions. The synthesized materials, WLP900 and WLP900CO_2_, exhibited enhanced textural and surface properties compared to carbon obtained from untreated walnut biomass, reflecting the influence of the liqueur preparation process and post-carbonization CO_2_ treatment. The BET analysis revealed that WLP900 possessed a slightly higher specific surface area (737 m^2^ g^−1^), while the CO_2_ activation in WLP900CO_2_ led to modest decreases in the surface area and pore volume, likely due to structural rearrangement. Despite these changes, the elemental analysis confirmed higher oxygen and nitrogen contents in WLP900CO_2_, contributing to distinct surface charge characteristics and adsorption behavior.

The adsorption kinetics followed a pseudo-second-order model, indicating chemisorption-like rate control, although the equilibrium data were well described by both the Freundlich and Langmuir isotherms, consistent with heterogeneous multilayer adsorption. The maximum adsorption capacity for WLP900CO_2_ reached 45.2 ± 0.2 mg g^−1^ at 30 °C. The thermodynamic analysis revealed that the adsorption was endothermic, spontaneous, and entropy-driven, with temperature-dependent improvements in performance more pronounced for WLP900CO_2_. This behavior reflects the enhanced desolvation of polar surface sites at elevated temperatures, facilitating stronger interactions between hydrophobic CHP molecules and the carbon surface.

The physicochemical characterization and adsorption data together suggest that CHP removal occurs predominantly via physisorption mechanisms, involving van der Waals forces and π–π interactions within micropores. WLP900 showed better adsorption at lower temperatures due to its higher surface area and greater density of acidic surface groups, while WLP900CO_2_ benefited from surface polarity and desolvation-enhanced site accessibility at elevated temperatures. Dynamic filtration experiments confirmed the applicability of both materials under flow conditions, maintaining performances comparable to those in the batch experiments.

Although ethanol-based regeneration resulted in a notable loss of capacity after the first cycle, likely due to partial pore blockage or kinetic entrapment in narrow pores, the residual capacity (~10 mg g^−1^) remained stable over five cycles, indicating promising reusability with optimized regeneration protocols. This study highlights the viability of walnut liqueur pomace-derived carbon materials as sustainable, low-cost adsorbents for pesticide remediation. These findings advance waste valorization strategies and contribute to the development of efficient sorbents for environmental cleanup. Future work should focus on regeneration enhancement, long-term field validation, and application to broader classes of pollutants.

## Figures and Tables

**Table 1:** Experimental setup for material preparation.

Material	Precursor	Pre-Treatment	Post-Treatment
W900	Young walnuts	/	/
WLP900	Walnut liqueur pomace	Ethanol and saccharose	/
WLP900CO_2_	Walnut liqueur pomace	Ethanol and saccharose	CO_2_

**Table 2:** Textural properties of synthesized carbon materials.

Sample	S_BET_ (m^2^ g^−1^)	V_tot_ (cm^3^ g^−1^)	d_av_/nm
W900	303	0.139	2.08
WLP900	737	0.332	1.93
WLP900CO_2_	652	0.301	1.95

**Table 3:** The elemental compositions of the investigated materials obtained using EDX.

Sample	C (at.%)	O (at.%)	N (at.%)	K (at.%)	Mg (at.%)	P (at.%)	Ca (at.%)	Na (at.%)	S (at.%)	Cl (at.%)	Si (at.%)	I (at.%)
WLP900	85.35	7.27	6.55	0.38	0.12	0.11	0.08	0.05	0.03	0.02	0.02	0.01
WLP900CO_2_	68.66	18.48	11.60	0.07	0.12	0.02	0.00	0.17	0.00	0.00	0.86	0.00

**Table 4:** Non-linear kinetic model parameters for 5 × 10^−5^ mol dm^−3^ CHP adsorption onto 1 mg cm^−3^ materials.

	WLP900	WLP900CO_2_
Pseudo-first-order model		
q_e_ (mg g^−1^)	11.1 ± 0.9	10 ± 1
k_1_ (min^−1^)	0.873 ± 0.007	0.67 ± 0.01
χ^2^	1.607	1.655
R^2^	0.915	0.899
Pseudo-second-order model		
q_e_ (mg g^−1^)	11.8 ± 0.3	10.9 ± 0.4
k_2_ (mg min^−1^ g^−1^)	0.089 ± 0.004	0.072 ± 0.003
χ^2^	0.528	0.641
R^2^	0.972	0.961
Elovich kinetic model		
A (mg g^−1^ min^−1^)	323 ± 1	90.6 ± 0.1
Β (g mg^−1^)	0.797 ± 0.002	0.746 ± 0.001
χ^2^	0.163	0.101
R^2^	0.991	0.994
Intraparticle diffusion model		
Phase I		
C (mg g^−1^)	0	0.63 ± 0.02
k_id_ (mg g^−1^ min^−0.5^)	6.785	3.7 ± 0.2
R^2^	/	0.891
Phase II		
C (mg g^−1^)	5.05 ± 0.01	7.19 ± 0.03
k_id_ (mg g^−1^ min^−0.5^)	1.68 ± 0.02	0.492 ± 0.002
R^2^	0.992	0.980
Phase III		-
C (mg g^−1^)	9.8 ± 0.8	-
k_id_ (mg g^−1^ min^−0.5^)	0.27 ± 0.06	-
R^2^	0.822	-

**Table 5:** Adsorption isotherm parameters for CHP on 1 mg cm^−3^ carbon materials at 20 °C, 25 °C, and 30 °C.

	WLP900			WLP900CO_2_		
T (°C)	20	25	30	20	25	30
Freundlich isotherm						
K_F_ ((dm^3^ mg^−1^)^1/n^)	5.83 ± 0.08	4.85 ± 0.05	4.82 ± 0.03	3.5 ± 0.5	3.84 ± 0.05	4.69 ± 0.06
n	3.71 ± 0.09	2.46 ± 0.06	2.41 ± 0.03	4.0 ± 0.4	2.44 ± 0.05	2.32 ± 0.05
χ^2^	4.416	7.886	4.853	2.308	4.619	8.934
R^2^	0.924	0.949	0.971	0.855	0.955	0.952
Langmuir isotherm						
K_L_ (dm^3^ mg^−1^)	0.103 ± 0.002	0.039 ± 0.002	0.035 ± 0.003	0.186 ± 0.001	0.034 ± 0.003	0.034 ± 0.001
q_max_ (mg g^−1^)	21.9 ± 0.1	40.1 ± 0.2	42.9 ± 0.2	11.2 ± 0.1	33.6 ± 0.3	45.2 ± 0.2
χ^2^	0.901	1.807	2.966	0.095	1.924	1.674
R^2^	0.985	0.988	0.982	0.994	0.981	0.991
Temkin isotherm						
K_T_ (dm^3^ mg^−1^)	2.41 ± 0.03	1.61 ± 0.09	2.2 ± 0.2	4.22 ± 0.04	1.63 ± 0.09	1.42 ± 0.01
b_T_ (J g mol^−1^ mg^−1^)	669 ± 5	436 ± 9	470 ± 30	1340 ± 50	542 ± 9	400 ± 10
χ^2^	1.275	13.832	19.587	50.61	10.070	19.547
R^2^	0.978	0.911	0.882	0.961	0.902	0.894
D-R isotherm						
q_DR_ (mg g^−1^)	18.8 ± 0.7	33 ± 4	34 ± 5	9.69 ± 0.09	27 ± 5	35 ± 1
K_DR_ (mol^2^ J^−2^)	(9.16 ± 0.08) × 10^−6^	(4.5 ± 0.5) × 10^−5^	(5.2 ± 0.5) × 10^−5^	(9.45 ± 0.09) × 10^−7^	(5.8 ± 0.6) × 10^−5^	(4.7 ± 0.2) × 10^−5^
E (J mol^−1^)	234 ± 7	110 ± 40	98 ± 6	728 ± 9	92 ± 6	100 ± 2
χ^2^	4.011	21.351	24.903	1.560	16.103	20.123
R^2^	0.931	0.863	0.850	0.902	0.843	0.891

**Table 6:** Maximum adsorption capacities for CHP adsorption onto different biochars and their textural properties.

Material	q_max_ (mg g^−1^)	S_BET_ (m^2^ g^−1^)	V_tot_ (cm^3^ g^−1^)	d_av_/nm	Reference
Pyrolyzed plum pomace	0.2	Not provided	Not provided	Not provided	[48]
Date palm biochar	7.0	Not provided	Not provided	Not provided	[49]
Ficus nitida biochar	12.5	Not provided	Not provided	Not provided	[49]
Cashew nutshell biochar	31.3	112	0.075	0.14	[50]
Wheat straw-derived biochar	16.0	467	0.260	2.19	[51]
WLP900	42.9	737	0.332	1.93	This work
WLP900CO_2_	45.2	652	0.301	1.95	This work

**Table 7:** Thermodynamic parameters of CHP adsorption onto WLP900 and WLP900CO_2_ at 20, 25, and 30 °C.

Material	T (oC)	ΔH^0^ (kJ mol^−1^)	ΔS^0^ (J mol^−1^ K^−1^)	ΔG^0^ (kJ mol^−1^)	R^2^
WLP900	20	46.7 ± 0.6	200 ± 5	−12.0 ± 0.6	0.971
	25			−13.0 ± 0.6	
	30			−14.0 ± 0.6	
WLP900CO_2_	20	101 ± 7	379 ± 8	−10.3 ± 0.7	0.987
	25			−12.2 ± 0.7	
	30			−14.1 ± 0.8	

## Data Availability

The original contributions presented in this study are included in the article. Further inquiries can be directed to the corresponding author.

## Supplementary Materials

The following supporting information can be downloaded at: https://www.mdpi.com/article/10.3390/ma18133072/s1, Table S1: The adsorption kinetics are described by the following equations. Table S2: The adsorption isotherms are described by the following equations; Figure S1: N_2_ adsorption isotherm of W900 sample; Figure S2: N_2_ adsorption isotherm of WLP900 sample; Figure S3: N_2_ adsorption isotherm of WLP900CO_2_ sample; Figure S4: (a) Cumulative specific surface vs. pore diameter plot; (b) specific surface area distribution plot; Figure S5: ATR-FTIR spectra of investigated materials; Figure S6. Influence of pH on the zeta potential of WLP900 and WLP900CO2 material suspensions; Figure S7: Van’t Hoff plots for CHP adsorption onto WLP900 and WLP900CO_2_ at 20, 25, and 30 °C; Figure S8: Regeneration and reuse of WLP900 and WLP900CO_2_ at 20 °C.
