# Supplementary material for: Application of Carbon Materials Derived from Nocino Walnut Liqueur Pomace Residue for Chlorpyrifos Removal from Water
Source: Materials (Basel). 2025 Jun 28;18(13):3072. doi: 10.3390/ma18133072 (PMC12251006; doi:10.3390/ma18133072)
Supplement: Supplementary file 1 [file materials-18-03072-s001.zip › materials-3602057-supplementary.pdf]

Supplementary Material

Application of Carbon Materials Derived from Nocino Walnut Liqueur Pomace Residue for Chlorpyrifos Removal from Water

Milena Zlatković [1], Rialda Kurtić [1], Igor A. Pašti [1,2], Tamara Tasić [3], Vedran Milanković [3], Nebojša Potkonjak [3], Christoph Unterweger [4] and Tamara Lazarević-Pašti [3,*]

[1] Faculty of Physical Chemistry, University of Belgrade, Studentski Trg 12–16, 11158 Belgrade, Serbia
[2] Serbian Academy of Sciences and Arts, Kneza Mihaila 35, 11000 Belgrade, Serbia
[3] VINČA Institute of Nuclear Sciences—National Institute of the Republic of Serbia, University of Belgrade, Mike Petrovica Alasa 12–14, 11000 Belgrade, Serbia
[4] Wood K Plus—Kompetenzzentrum Holz GmbH, Altenberger Strasse 69, 4040 Linz, Austria
* Correspondence: tamara@vin.bg.ac.rs

Table S1. The adsorption kinetics are described by the following equations.

Pseudo-first-order model	$q_t = q_e(1 - e^{-k_1 t})$	(S1)
Pseudo-second-order model	$q_t = \frac{q_e^2 k_2 t}{1 + q_e k_2 t}$	(S2)
Elovich kinetic model	$q_t = \frac{1}{\beta}(1 + \alpha\beta t)$	(S3)
Intraparticle diffusion model	$q_t = k_{id} t^{0.5} + C$	(S4)

In these equations, q_t represents the amount of adsorbate adsorbed at time (t) (mg g^{-1}), and q_e represents the equilibrium adsorption capacity (mg g^{-1}). The constants k_1 (min^{-1}) and k_2 (g mg^{-1} min^{-1}) are the rate constants for the pseudo-first-order and pseudo-second-order models, respectively. The Elovich model parameters include α, which signifies the initial adsorption rate (mg g^{-1} min^{-1}), and β, which signifies the desorption constant (g mg^{-1}). The intraparticle diffusion model uses k_{id} as the adsorption rate constant (mg g^{-1} min$^{-0.5}$) and C as a boundary-layer parameter (mg g^{-1}).

Table S2. The adsorption isotherms are described by the following equations.

Freundlich model	$q_e = K_F C_e^{\frac{1}{n}}$	(S5)
Langmuir model	$q_e = \frac{q_{max} K_L C_e}{1 + K_L C_e}$	(S6)

Temkin model	$q_e = \frac{RT}{b_T} lnK_T C_e$	(S7)
Dubinin–Radushkevic model	$q_e = q_{DR} e^{-K_{DR}\varepsilon^2}$	(S8)

In these equations, q_e represents the amount adsorbed at equilibrium (mg g^{-1}), while C_e represents the equilibrium adsorbate concentration (mg dm^{-3}). In the Freundlich isotherm model, K_F ((dm^3 $mg^{-1})^{1/n}$) and n are constants describing the adsorption capacity and intensity, respectively. The Langmuir model uses K_L (dm^3 mg^{-1}) as the Langmuir constant and q_{max} (mg g^{-1}) as the theoretical maximum adsorption capacity of the monolayer. For the Temkin isotherm, b_T (J g mol^{-1} mg^{-1}) and K_T (dm^3 mg^{-1}) are constants related to the heat of the adsorption and equilibrium binding. The Dubinin–Raduskevic isotherm defines q_{DR} (mg g^{-1}) as the theoretical saturation capacity and K_{DR} (mol^2 J^{-2}) as the constant associated with the mean free energy per mole of adsorbent ($\varepsilon = RT \times \ln(1 + 1/C_e)$).

Thermodynamic parameters, including the enthalpy (ΔH), entropy (ΔS), and Gibbs free energy (ΔG), were evaluated to gain a deeper understanding of the interactions between contaminant molecules and the surface of the adsorbent material.

The values of the ΔH^0 and ΔS^0 were obtained as the intercept and slope of the Van't Hoff equation plot (Equation S9), where the standard Gibbs free energy change is expressed as $\Delta G^0 = -RTlnK^0_{dist}$.

The standard distribution coefficient was determined using Equation S10. To ensure it was dimensionless, the ratio $\frac{q_e}{C_e}$ was multiplied by C^0 and q^0, which correspond to the standard states of the contaminant in solution (1 mol dm^{-3}) and in the adsorbed state (1 mol kg^{-1}), respectively:

$$lnK^0_{dist} = -\frac{\Delta H^0}{RT} + \frac{\Delta S^0}{R} \quad \text{(S9)}$$

$$K^0_{dist} = \frac{q_e}{C_e} \times \frac{C^0}{q^0} \quad \text{(S10)}$$

Finally, the Gibbs free energy was also determined using the Gibbs–Helmholtz equation (Equation S11):

$$\Delta G^0 = \Delta H^0 - T\Delta S^0 \quad \text{(S11)}$$

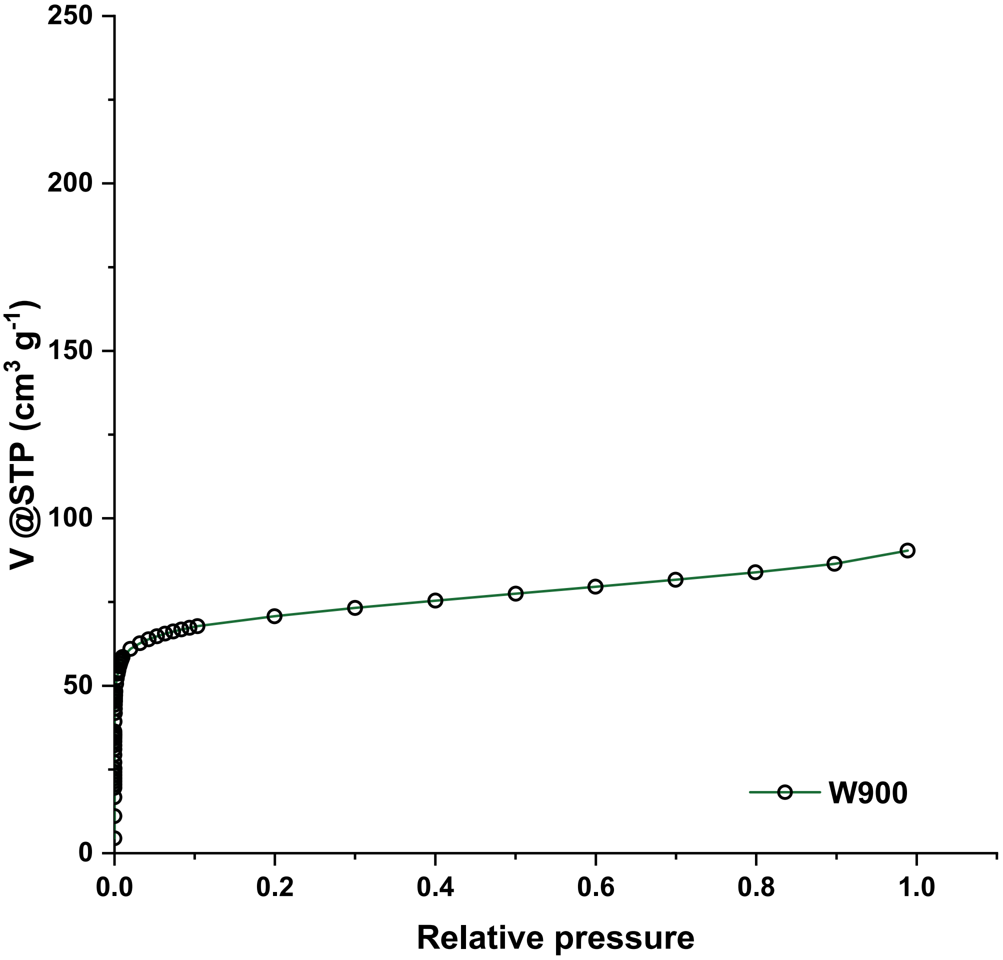

Figure S1. N_2 adsorption isotherm of W900 sample.

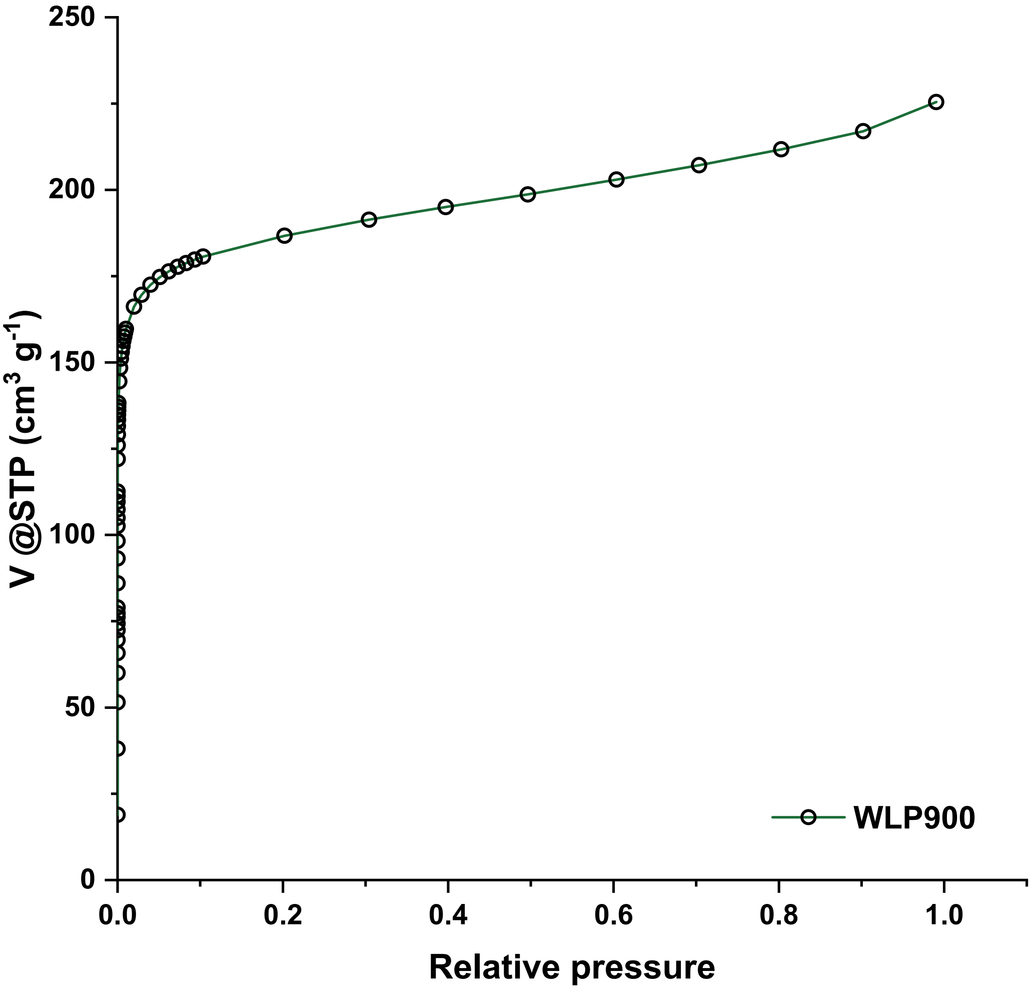

Figure S2. N_2 adsorption isotherm of WLP900 sample.

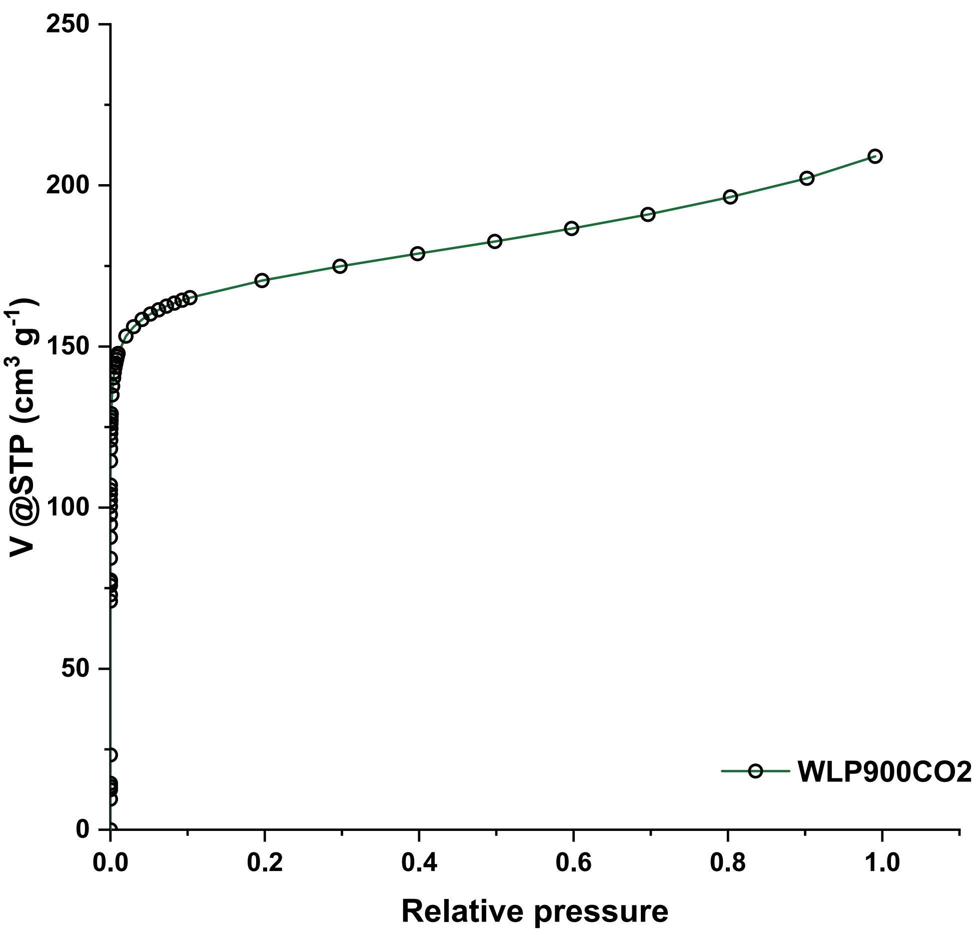

Figure S3. N_2 adsorption isotherm of WLP900CO_2 sample.

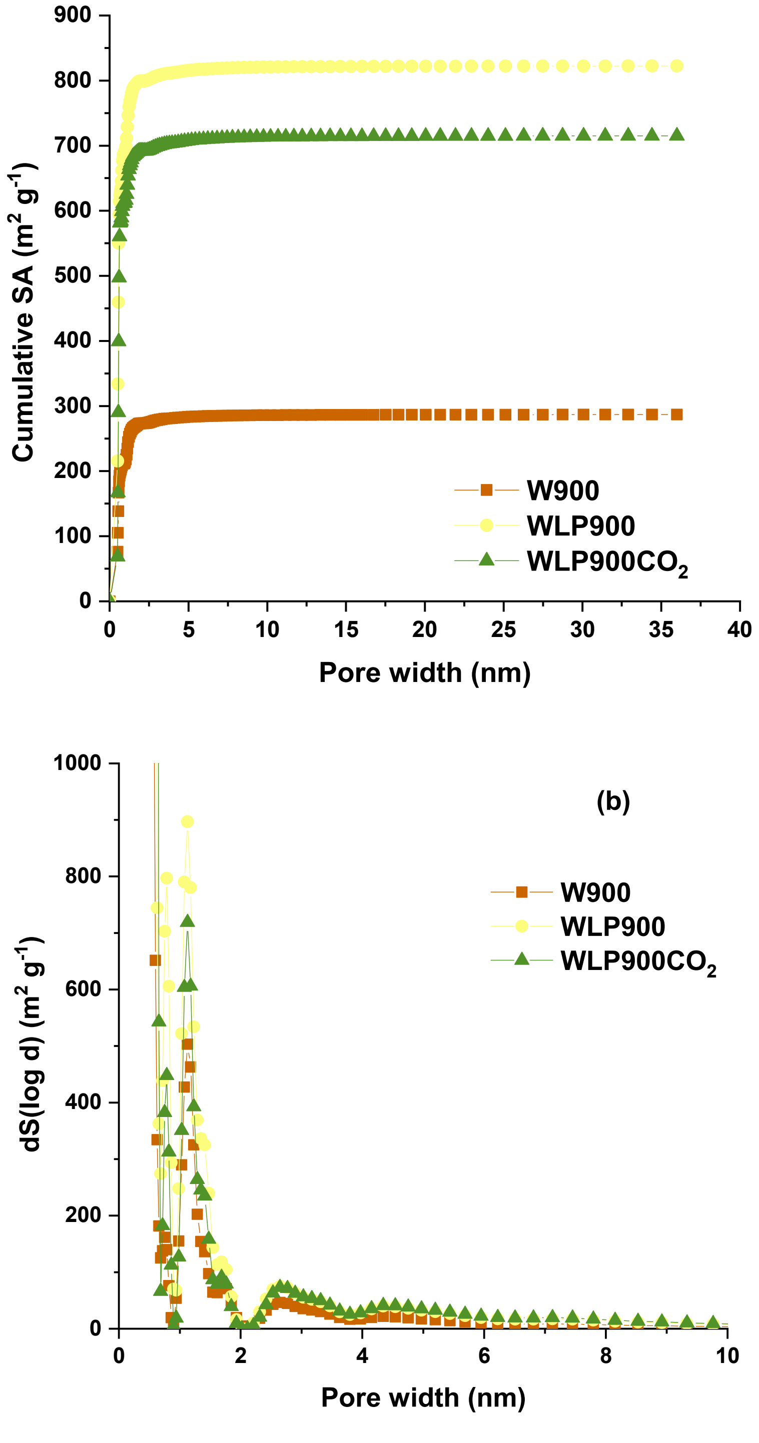

Figure S4: (a) Cumulative specific surface vs. pore diameter plot; (b) specific surface area distribution plot.

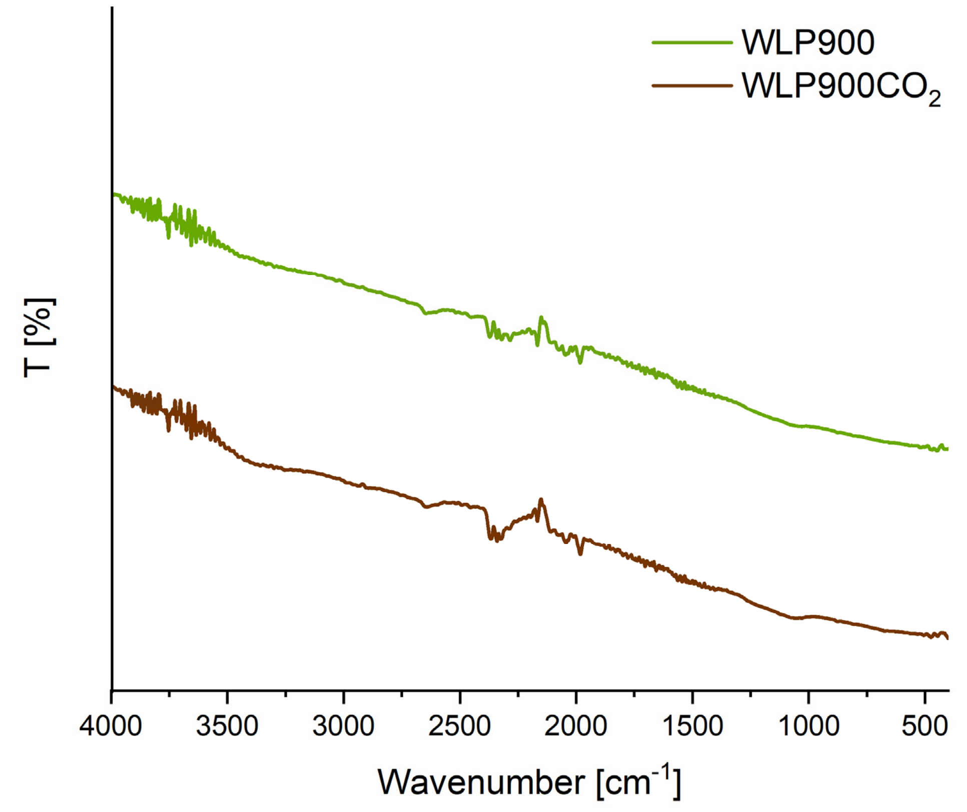

Figure S5. ATR-FTIR spectra of investigated materials.

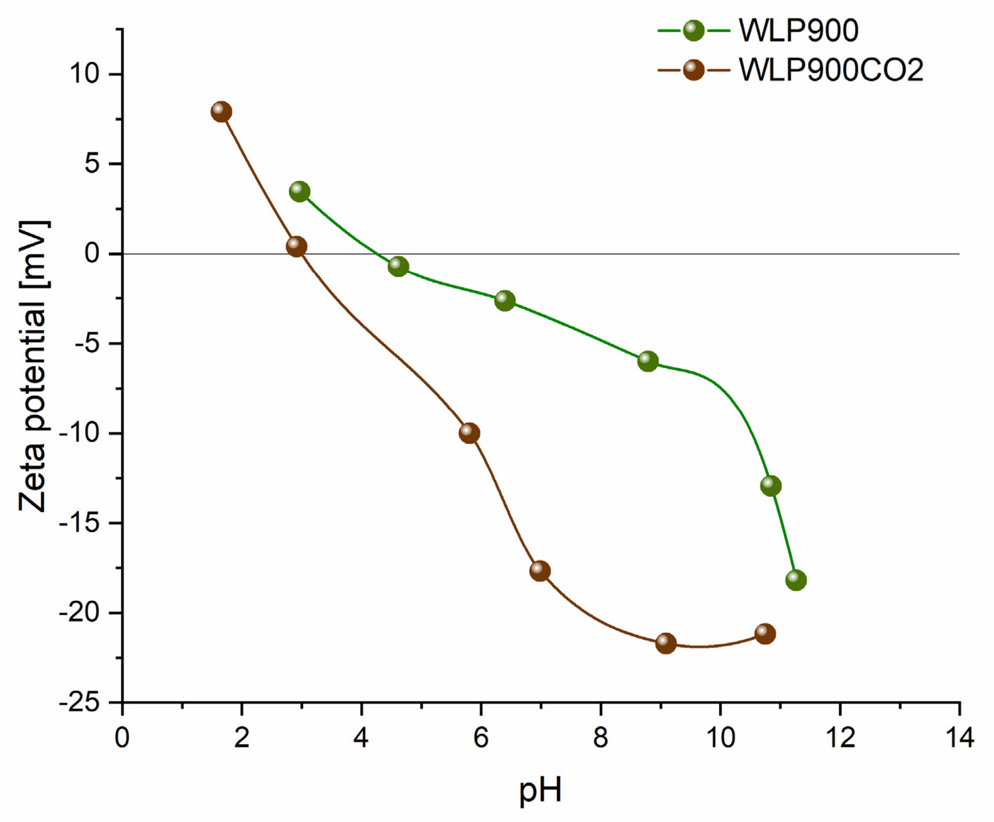

Figure S6. Influence of pH on the zeta potential of WLP900 and WLP900CO_2 material suspensions.

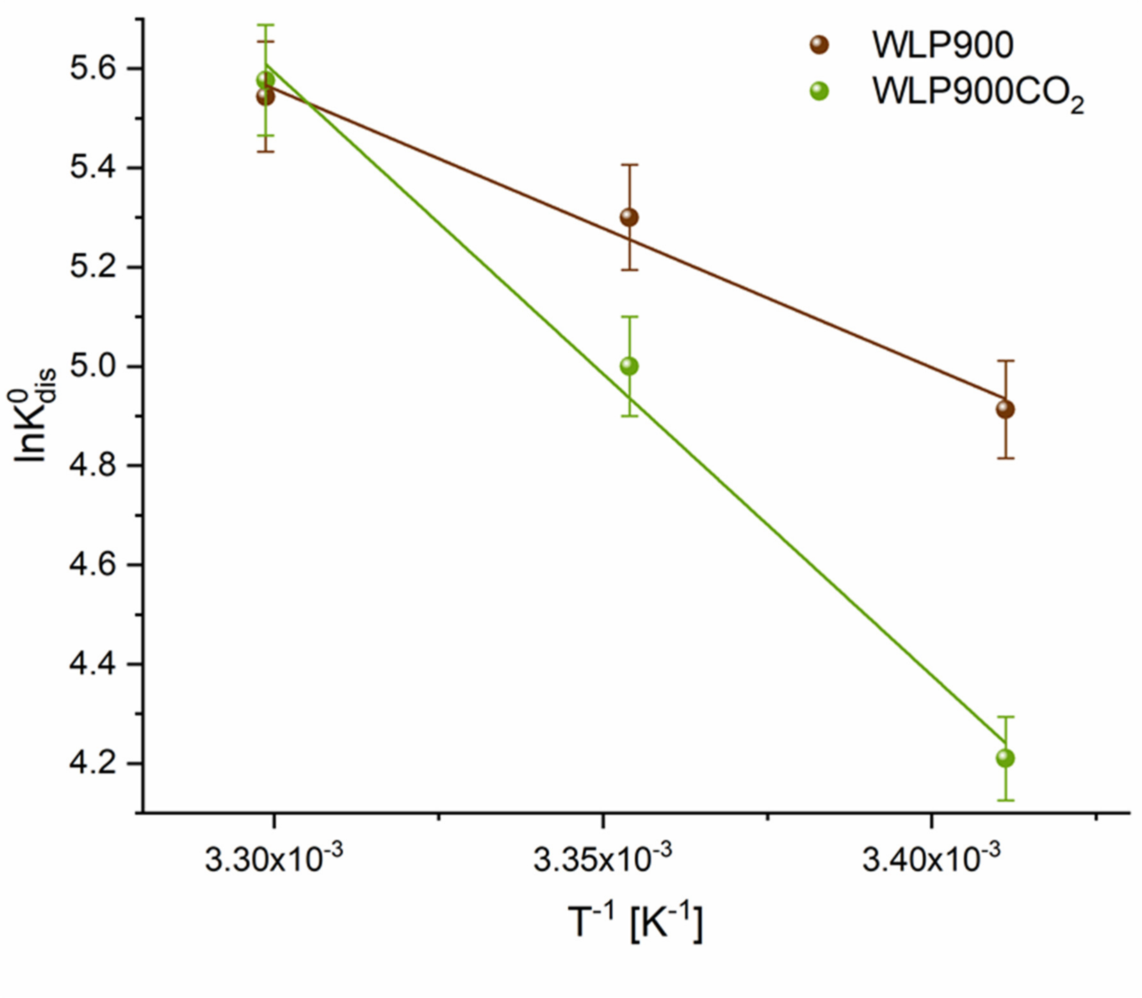

Figure S7. Van't Hoff plots for CHP adsorption onto WLP900 and WLP900CO_2 at 20, 25, and 30 °C.

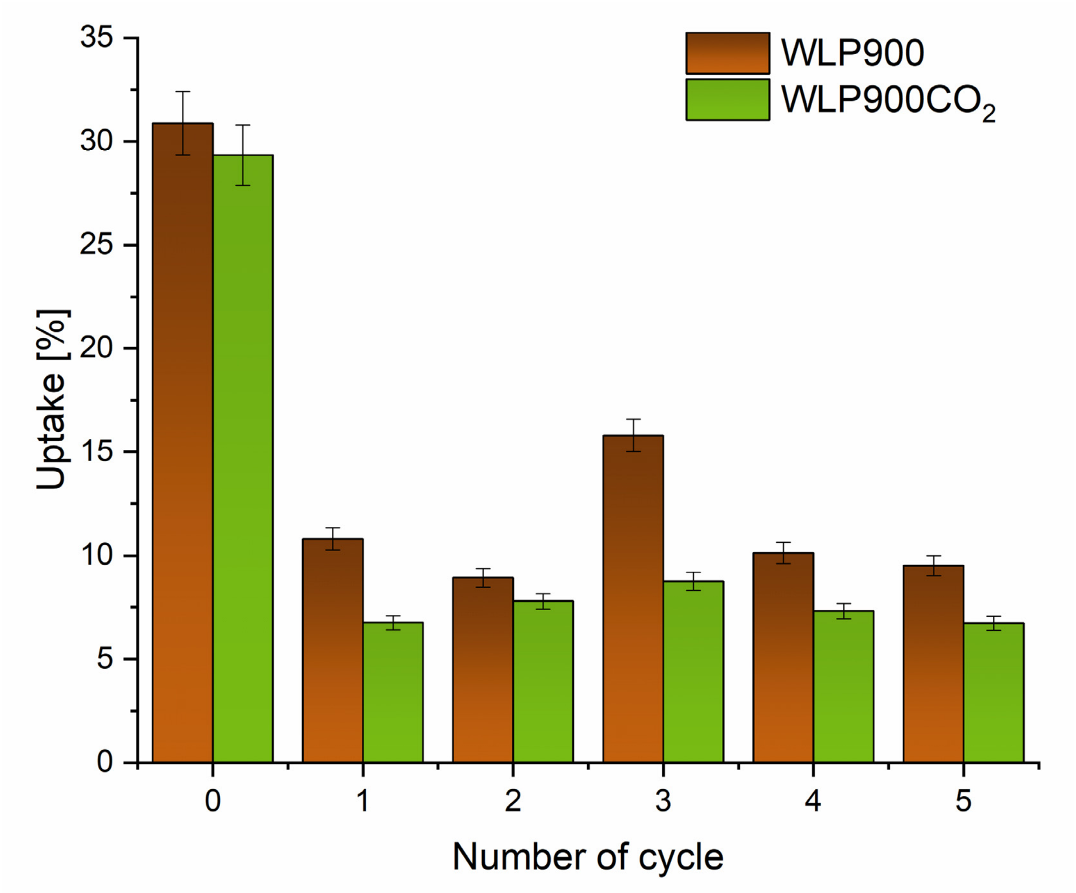

Figure S8. Regeneration and reuse of WLP900 and WLP900CO_2 at 20°C.
